# Protective Effect of Peroxisome Proliferator-Activated Receptor *α* Activation against Cardiac Ischemia-Reperfusion Injury Is Related to Upregulation of Uncoupling Protein-3

**DOI:** 10.1155/2016/3539649

**Published:** 2015-12-07

**Authors:** Jong Wook Song, Hyo Jung Kim, Hyelin Lee, Jae-woo Kim, Young-Lan Kwak

**Affiliations:** ^1^Department of Anesthesiology and Pain Medicine, Yonsei University College of Medicine, Seoul 03722, Republic of Korea; ^2^Anesthesia and Pain Research Institute, Yonsei University College of Medicine, Seoul 03722, Republic of Korea; ^3^Department of Biochemistry and Molecular Biology, Integrated Genomic Research Center for Metabolic Regulation, Institute of Genetic Science, Yonsei University College of Medicine, Seoul 03722, Republic of Korea; ^4^Brain Korea 21 PLUS Project for Medical Science, Yonsei University, Seoul 03722, Republic of Korea; ^5^Severance Biomedical Science Institute, Yonsei University College of Medicine, Seoul 03722, Republic of Korea

## Abstract

Activation of peroxisome proliferator-activated receptor *α* (PPAR*α*) confers cardioprotection, while its mechanism remains elusive. We investigated the protective effect of PPAR*α* activation against cardiac ischemia-reperfusion injury in terms of the expression of uncoupling protein (UCP). Myocardial infarct size and UCP expression were measured in rats treated with WY-14643 20 mg/kg, a PPAR*α* ligand, or vehicle. WY-14643 increased UCP3 expression *in vivo*. Myocardial infarct size was decreased in the WY-14643 group (76 ± 8% versus 42 ± 12%, *P<0.05*). During reperfusion, the incidence of arrhythmia was higher in the control group compared with the WY-14643 group (9/10 versus 3/10, *P<0.05*). H9c2 cells were incubated for 24 h with WY-14643 or vehicle. WY-14643 increased UCP3 expression in H9c2 cells. WY-14643 decreased hypoxia-stimulated ROS production. Cells treated with WY-14643 were more resistant to hypoxia-reoxygenation than the untreated cells. Knocking-down UCP3 by siRNA prevented WY-14643 from attenuating the production of ROS. UCP3 siRNA abolished the effect of WY-14643 on cell viability against hypoxia-reoxygenation. In summary, administration of PPAR*α* agonist WY-14643 mitigated the extent of myocardial infarction and incidence of reperfusion-induced arrhythmia. PPAR*α* activation conferred cytoprotective effect against hypoxia-reoxygenation. Associated mechanisms involved increased UCP3 expression and resultant attenuation of ROS production.

## 1. Introduction

Peroxisome proliferator-activated receptor *α* (PPAR*α*), a member of the nuclear hormone receptor superfamily, is a transcription factor that regulates the expression of genes involved in cellular lipid metabolism [1]. Myocardial PPAR*α*-overexpression increased fatty acid uptake and oxidation with a concomitant decrease in glucose uptake and oxidation [2, 3], and these metabolic changes were associated with left ventricular hypertrophy and systolic dysfunction [4]. In contrast, pharmacologic activation of PPAR*α* has been shown to confer myocardial protection against acute ischemia-reperfusion injury [5–7]. However, the underlying mechanism of cardioprotection by acute PPAR*α* activation remains unclear.

Uncoupling proteins (UCPs) are inner mitochondrial carrier proteins that induce proton leak and dissipate the mitochondrial electrochemical gradient [8]. UCP1 was firstly discovered as a regulator of thermogenesis in brown adipose tissue. UCP2 and UCP3 were found to be expressed in various tissues including the heart while their role in the heart is still elusive. Previous studies suggested that UCPs may have a protective role during oxidative stress. Mitochondrial reactive oxygen species (ROS) generation is known to be proportional to electrochemical gradient across the inner membrane [9]. Mild uncoupling and decreased proton gradient across the mitochondrial inner membrane reduced ROS production [10]. In conjunction, dinitrophenol, a pharmacologic uncoupling agent, exerted cardioprotective effect [11, 12]. Overexpression of UCP2 in cardiomyocyte attenuated ROS production and increased tolerance to oxidative stress [13]. Moreover, UCP2 and UCP3 were upregulated after ischemic preconditioning [14].

UCP3 is upregulated by circulating free fatty acid and PPAR*α* has been shown to be a mediator of transcriptional activation of UCP3 [15, 16]. Based on the regulatory role of PPAR*α* in the expression of UCP, we hypothesized that the mechanism of cardioprotective effect of PPAR*α* against ischemia-reperfusion injury could involve increased expression of UCP, particularly UCP3, and resultant attenuation of ROS generation. This study aimed to investigate whether WY-14643, a PPAR*α* ligand, conferred protection against acute myocardial ischemia-reperfusion injury, and the cardioprotection involved upregulation of UCP3 and reduced ROS production.

## 2. Materials and Methods

### 2.1. Animal Preparation and Experimental Protocol

This study was approved by the institutional ethics committee for laboratory animal experiments and all experiments were conducted according to the Guide for the Care and Use of Laboratory Animals (National Institutes of Health publication number 85-23, revised 1996).

Male Sprague-Dawley rats weighing 250 to 350 g were anesthetized with sodium pentobarbital 50 mg/kg i.p. bolus. Additional intermittent bolus of sodium pentobarbital 10 mg/kg every 1 h was followed for the maintenance of anesthesia. The left jugular vein was cannulated for delivery of fluid and Patent Blue dye. The left carotid artery was cannulated for continuous monitoring of mean arterial pressure (MAP) and heart rate (HR). A 3-lead electrocardiogram was placed for detection of ischemic change and arrhythmia. Premature ventricular beats, ventricular tachycardia, and ventricular fibrillation were evaluated according to the criteria of the Lambeth convention [17]. The animals were ventilated via tracheostomy with 60% oxygen/air mixture at a tidal volume of 8 mL/kg. Respiratory rate was initially set to 50 breaths/min and adjusted to maintain the arterial PCO_2_ between 35 and 40 mmHg. The heart was exposed via left thoracotomy and a snare was placed around the left anterior descending coronary artery (LAD). After the surgical preparation, all rats were stabilized for 30 min before LAD occlusion. Ischemia was induced by tightening the snare. Ischemia was confirmed by visual inspection of pale color on the anterior wall of the heart and ST segment elevation on electrocardiogram. After 30 min of ischemia, myocardium was reperfused by loosening the snare for 2 h.

Animals were randomly allocated into two groups. The WY group received PPAR*α* agonist WY-14643 (4-chloro-6-[2,3-xylidino]-2-pyrimidinylthioacetic acid, Sigma-Aldrich Korea, Seoul, Korea) 20 mg/kg i.p. 4 h before LAD occlusion for the measurement of infarct size, or 4 h before the excision of heart for RT-PCR and western blot analysis. The control group received the same volume of 5% dimethyl sulfoxide (DMSO).

### 2.2. Measurement of Infarct Size

The LAD was reoccluded after 2 h of reperfusion and 2 mL/kg of 10% Patent Blue was administered via the left jugular vein. The heart was rapidly extracted and the right ventricle was carefully removed. The left ventricle was incubated at −20°C for 20 min. Thereafter, the left ventricle was sectioned into 1–1.5 mm slices perpendicular to the apex-base axis. Then the slices were incubated in 2% triphenyltetrazolium chloride for 30 min at 37°C to distinguish the infarcted tissue from the viable myocardial areas. Areas which were not stained by Patent Blue indicated area at risk. Pale discolored areas represented infarcted myocardium while viable myocardial areas were stained red. Both planes of the slices were photographed and infarct size was calculated as area of infarction/area at risk × 100 (%).

### 2.3. Cell Culture

The H9c2 cell line was obtained from ATCC (Manassas, VA, USA). The cells were cultured in ATCC-formulated Dulbecco's Modified Eagle's Medium containing 10% fetal bovine serum at 37°C in an atmosphere of 95% air and 5% CO_2_, based on the manufacturer's recommendations. The cells were treated with WY-14643 or vehicle for 24 h before RT-PCR, western blot, ROS measurement, or cell survival experiment. For cellular hypoxia-reoxygenation, cells were incubated for 20 h in an anoxic chamber immediately after the culture medium was exchanged with the medium that was preincubated for 8 h in an anoxic chamber and reoxygenated for 2 h with normal medium.

### 2.4. Gene Silencing with siRNA

Cells were seeded into 100-mm culture dishes 18–24 h prior to transfection. Cells were transfected with 80 nM control siRNA (Ambion, Austin, TX, USA) and UCP3 siRNA (Genolution, Seoul, Korea), in serum-free medium using RNAiMAX (Invitrogen, Carlsbad, CA, USA). Following incubation for 6 h, the transfection medium was replaced with fresh medium, and the cells were incubated for additional 48 h, at which point they were treated with the indicated reagents for the indicated time.

### 2.5. RT-PCR

Total RNA was isolated from cultured cells using TRIzol (Invitrogen, Carlsbad, CA, USA) according to the manufacturer's instructions. First-strand cDNA synthesis from 5 *μ*g of total RNA was performed using SuperScript II reverse transcriptase (Invitrogen, Carlsbad, CA, USA) primed by random hexamer primer. The transcripts of UCP2, UCP3, and GAPDH were evaluated by PCR analysis. The PCR amplification was performed in a final reaction volume of 50 *μ*L under the following conditions: preincubation for 5 min at 94°C, 30 cycles of 30 s at 94°C, 30 s at 58°C, and 45 s at 72°C, and one final incubation for 10 min at 72°C. The PCR products were separated by electrophoresis through 1.5% agarose gels. For quantitative RT-PCR, an aliquot (1/40) of the reaction was used for quantitative PCR using the SYBR Green PCR Master Mix (Applied Biosystems, Life Technologies Korea, Seoul, Korea) and gene-specific primers. RT-PCR products were quantified using the ABI PRISM 7300 RT-PCR System (Applied Biosystems, Life Technologies Korea, Seoul, Korea). RT-PCR was performed using the following primers: UCP2, 5′-CTCCC AATGT TTGCC CGAAA-3′, 5′-ACTGG CCCAA GGCAG AGTTC-3′; UCP3, 5′-TATGG TGCGC ACAGA GGGTC-3′, 5′-CACCA CATCC GTGGG TTGAG-3′; GAPDH, 5′-ACCAC AGTCC ATGCC ATCAC-3′, 5′-TCCAC CACCC TGTTG CTGTA-3′. GAPDH mRNA was also measured as an invariant control.

### 2.6. Western Blot Analysis

Cells treated with the indicated reagents were washed in ice-cold PBS and lysed in PRO-PREP Protein Extraction Solution (iNtRON Biotechnology, Seoul, Korea). An aliquot of the cell lysate was subjected to SDS-polyacrylamide gel electrophoresis and transferred onto Hybond-P polyvinylidene difluoride membrane (Amersham Biosciences, Corston, UK). Membranes blocked with 5% nonfat milk in Tris-Buffered Saline (TBS) containing 0.1% Tween-20 overnight at 4°C were reacted with the indicated specific antibodies in TBS containing 1% BSA and 0.05% Tween-20 overnight at 4°C and then incubated with peroxidase-conjugated goat antibody diluted to 1 : 3000 for 2 h at room temperature. After extensive washing in TBS containing 0.1% BSA and 0.1% Tween-20, immunoreactive bands were detected using West-ZOL Plus (iNtRON Biotechnology, Seoul, Korea).

### 2.7. MTT Assay

MTT (3-[4,5-dimethylthiazol-2-yl]-2,5 diphenyl tetrazolium bromide, final concentration of 0.1 mg/mL) was added to the culture medium, and cells were incubated for additional 4 h. The medium was removed and formazan crystals formed by the reduction of MTT by mitochondrial dehydrogenases in living cells were solubilized in acidified isopropanol and measured spectrophotometrically at 570 nm.

### 2.8. Measurement of Reactive Oxygen Species

After the cells were subjected to hypoxia or normoxia as described above, they were incubated with 5 *μ*M 2′,7′-dichlorofluorescein diacetate (DCF-DA, Calbiochem, San Diego, CA, USA) for a final 30 min, then harvested, and washed twice with ice-cold PBS. The cells were immediately analyzed for fluorescence intensity using a FACSCalibur flow cytometer (Becton-Dickinson Immunocytometry Systems, San Jose, CA, USA) and CellQuestPro software.

Intracellular superoxide production was measured by lucigenin-amplified chemiluminescence or a fluorescent indicator, dihydroethidium (DHE). To determine the level of superoxide anion by lucigenin, cells were treated with each reagent and, then, trypsinized and collected by centrifugation at 1,000 rpm for 5 min. After washing twice with ice-cold PBS, the cells were resuspended in HBSS buffer (5.4 mM KCl, 0.3 mM Na_2_HPO_4_, 0.4 mM KH_2_PO_4_, 4.2 mM NaHCO_3_, 1.3 mM CaCl_2_, 0.5 mM MgCl_2_, 0.6 mM MgSO_4_, 137 mM NaCl, and 5.6 mM glucose, pH 7.4) containing 5 mM of lucigenin. The lucigenin-derived chemiluminescence was determined every 50 s for a total of 5 min, by LB96V luminometer (EG&G Berthold, Bad Wildbad, Germany). For analysis of intracellular superoxide production, cells treated as described above were washed with ice-cold PBS and incubated for 30 min at 37°C with 10 mM of DHE in PBS. Following incubation in a humidified chamber protected from light, the red fluorescence was detected through a 580 nm longpass filter using a fluorescence microscope (Olympus, Tokyo, Japan).

Mitochondrial superoxide was detected by MitoSOX Red-based flow cytometry. After the cells were subjected to hypoxia or normoxia as described above, they were incubated with 4 *μ*M MitoSOX Red superoxide indicator (Invitrogen) for 30 min at 37°C. After centrifugation, the cell pellets were resuspended and washed once at room temperature phenol red-free, HEPES-buffered DMEM supplemented with 2% dialyzed FBS. The cells were then resuspended in 750 *μ*L of the same buffer containing 1 *μ*g/mL DAPI and kept on ice until flow cytometric analysis.

### 2.9. Statistical Analysis

Values are expressed as mean ± SD. Data were analyzed by independent *t*-test except the hemodynamic variables, which were analyzed by repeated measures of ANOVA. The incidence of arrhythmia was compared by chi-square test. SAS (version 9.1.3, SAS Institute, Inc., Cary, NC, USA) was used for statistical analysis. A *P* value of less than 0.05 was considered statistically significant.

## 3. Results

### 3.1. Myocardial Infarct Size following LAD Occlusion

**Figure 1:**
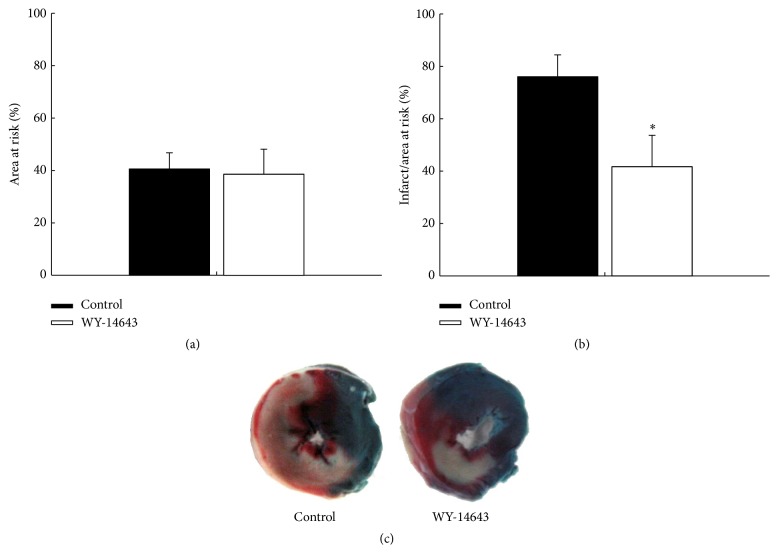
WY-14643 decreased myocardial infarct size. WY-14643 20 mg/kg was administered 4 h before the occlusion of left anterior descending coronary artery. (a) Area at risk was similar between the groups. (b) WY-14643 decreased the percentage of myocardial infarct size to area at risk. ^*∗*^ *P* < 0.05 versus control (*n* = 10). (c) Representative pictures of stained myocardial sections.

Area at risk was similar between the groups (41 ± 6% versus 39 ± 10%, control versus WY-14643, resp., *P* > 0.05, Figure 1(a)). Myocardial infarct size was significantly smaller in the WY-14643 group compared with the control group (76 ± 8% versus 42 ± 12%, *P* < 0.05, Figure 1(b)).

### 3.2. Effect of WY-14643 on UCP Expression* In Vivo*

**Figure 2:**
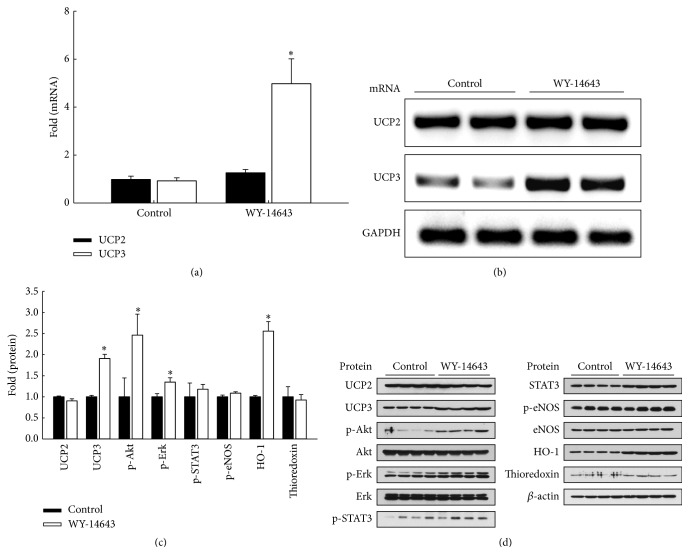
WY-14643 increased UCP3 expression in rats. WY-14643 20 mg/kg was administered 4 h before the extraction of heart. (a) Quantitative PCR shows that WY-14643 increased mRNA levels compared with vehicle. ^*∗*^ *P* < 0.05 (*n* = 3). (b) RT-PCR shows increased UCP3 mRNA expression following WY-14643 administration. (c) Western blot shows increased expression of UCP3 and HO-1, and phosphorylation of Akt and Erk after WY-14643 administration. Band intensities were quantified using an image analyzer and normalized by expression levels of *β*-actin. ^*∗*^ *P* < 0.05 (*n* = 4). (d) Representative blots are shown.

Animals received WY-14643 20 mg/kg i.p. 4 h before excision of the heart. UCP3 mRNA expression was significantly increased in the WY-14643 group compared with the control group (*P* < 0.05, Figures 2(a)-2(b)). UCP3 protein level was increased with WY-14643 administration, and heme oxygenase-1 (HO-1) expression, and phosphorylated Akt and Erk were also increased (*P* < 0.05, Figure 2(c)). UCP2 expression did not increase after WY-14643 administration.

### 3.3. Hemodynamic Variables and Incidence of Arrhythmia

Hemodynamic variables including mean arterial pressure, heart rate, and rate pressure product showed no significant differences between the groups (Table 1). During reperfusion, incidence of arrhythmia was significantly higher in the control group compared with the WY-14643 group (9/10 versus 3/10, *P* < 0.05). In the control group, ventricular tachycardia was detected in 2 rats and premature ventricular beats were detected in 7 rats. In the WY-14643 group, ventricular tachycardia was detected in one rat and premature ventricular beats were detected in 2 rats.

### 3.4. Effect of WY-14643 on UCP Expression* In Vitro*

**Figure 3:**
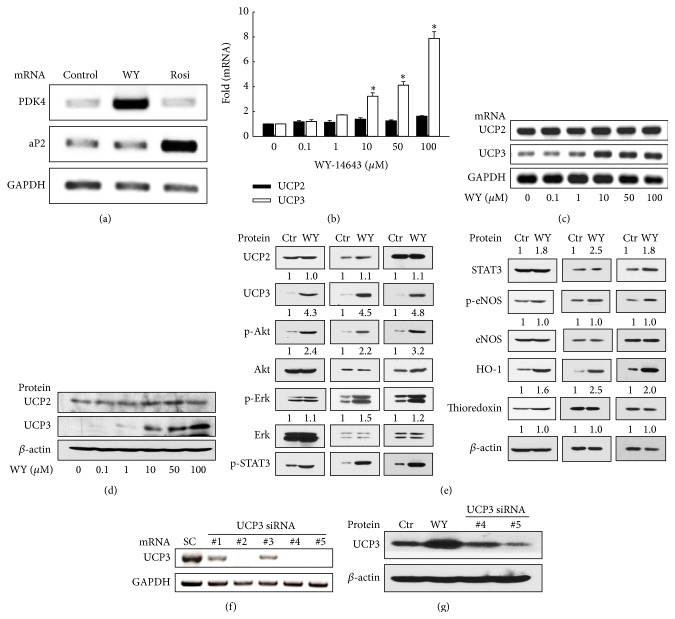
WY-14643 increased UCP3 expression in H9c2 cells. (a) Treatment of WY-14643 at 50 *μ*M increased expression of PPAR*α* target gene PDK4 mRNA and expression of PPAR*γ* target gene aP2 was not altered after the treatment of WY-14643. (b) Quantitative PCR shows that WY-14643 treatment at 10, 50, and 100 *μ*M for 24 h increased mRNA levels of UCP3 compared with vehicle treatment. ^*∗*^ *P* < 0.05 versus WY-14643 0 *μ*M (*n* = 3). (c) RT-PCR shows increased UCP3 mRNA expression following WY-14643 treatment. (d) Western blot of UCP3 shows increased protein level after WY-14643 treatment. (e) Treatment of WY-14643 at 50 *μ*M also increased phosphorylation of Akt and STAT3 and protein level of HO-1 as well as UCP3. Representative blots from three independent experiments are shown. Band intensities were quantified using an image analyzer, normalized by expression levels of *β*-actin, and fold changes are plotted under each band. (f) Transfection of UCP3 siRNA silenced UCP3 mRNA (#2, #4, and #5). (g) Western blot of UCP3 shows that transfection of UCP3 siRNA downregulated UCP3 protein level. #5 was used for further experiment. WY: WY-14643. Rosi: rosiglitazone. HO-1: heme oxygenase-1. SC: scramble.

Figure 3 illustrates the expression of UCP2 and UCP3 in H9c2 cell line after 24 h incubation in the control medium or experimental media containing 0.1 to 100 *μ*M of WY-14643. In cells treated with 50 *μ*M of WY-14643, expression of PDK4 mRNA, a PPAR*α* target gene, was increased. Expression of PPAR*γ* target gene aP2 was not altered after treatment with WY-14643, indicating that WY-14643 activated PPAR*α* specifically (Figure 3(a)). Upregulation of UCP3 mRNA was observed in cells treated with 10 *μ*M or greater concentration of WY-14643 compared with the control (*P* < 0.05, Figures 3(b)-3(c)). UCP3 protein increased after treatment of 10 *μ*M or greater concentration of WY-14643 (Figure 3(d)). The expression of UCP2 was not altered by WY-14643 treatment. Subsequently, whether WY-14643 activated other cardioprotective signaling pathways was examined. Treatment of WY-14643 at 50 *μ*M increased phosphorylation of Akt and STAT3 and protein level of HO-1 as well as UCP3 (Figure 3(e)). Transfection of UCP3 siRNA silenced UCP3 mRNA (Figure 3(f)), and UCP3 siRNA downregulated UCP3 protein level (Figure 3(g)).

### 3.5. WY-14643 Decreased Hypoxia-Stimulated ROS Production

**Figure 4:**
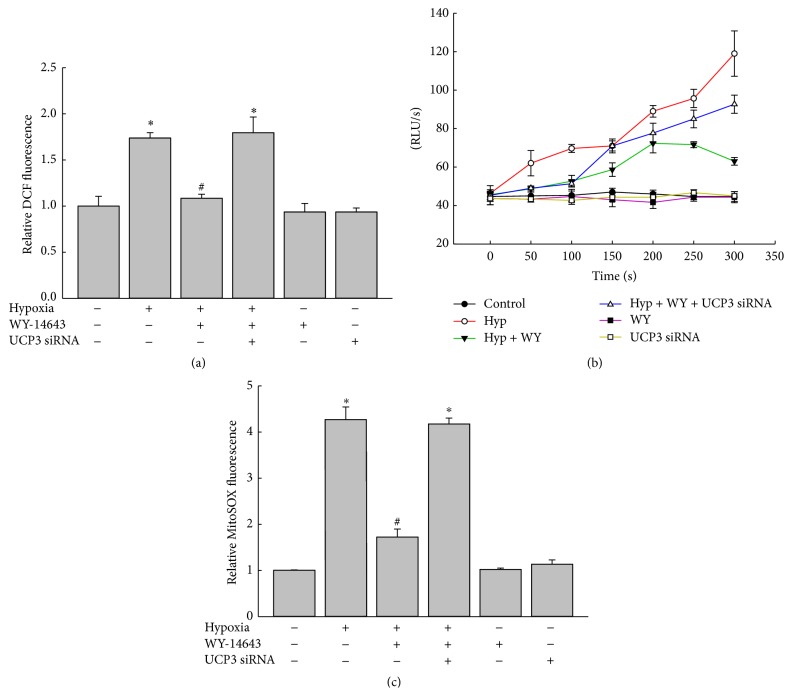
WY-14643 decreased hypoxia-stimulated intracellular ROS production. (a) Cells treated with 50 *μ*M of WY-14643 or vehicle were subjected to 20 h of hypoxia and ROS level was measured by DCF fluorescence. Hypoxia increased ROS generation and WY-14643 attenuated hypoxia-induced increase in ROS. UCP3 siRNA abrogated the effect of WY-14643 on ROS production. ^*∗*^ *P* < 0.05 versus control. ^#^ *P* < 0.05 versus hypoxia (*n* = 3). (b) Cells treated with 50 *μ*M of WY-14643 or vehicle were subjected to 20 h of hypoxia and ROS level was measured by lucigenin-amplified chemiluminescence. Hypoxia increased ROS generation and WY-14643 attenuated hypoxia-induced increase in ROS. UCP3 siRNA abrogated the effect of WY-14643 on ROS production (*n* = 3). (c) Cells treated with 50 *μ*M of WY-14643 or vehicle were subjected to 20 h of hypoxia and mitochondrial superoxide was measured by MitoSOX Red fluorescence. Hypoxia increased mitochondrial superoxide generation and WY-14643 attenuated hypoxia-induced increase in mitochondrial superoxide. UCP3 siRNA abrogated the effect of WY-14643 on mitochondrial superoxide production. ^*∗*^ *P* < 0.05 versus control. ^#^ *P* < 0.05 versus hypoxia (*n* = 3). Hyp: hypoxia; WY: WY-14643 treatment.

Assessing whether WY-14643 could reduce oxidative stress and UCP3 upregulation was involved in this process, cells treated with 50 *μ*M of WY-14643 or vehicle were subjected to 20 h of hypoxia, and ROS level was measured by DCF florescence. Hypoxia increased ROS generation (*P* < 0.05 versus control) and WY-14643 attenuated hypoxia-induced increase in ROS (*P* < 0.05 versus hypoxia). In cells transfected with UCP3 siRNA, WY-14643 treatment did not reduce hypoxia-induced ROS generation (*P* < 0.05 versus control, Figure 4(a)). Lucigenin-amplified chemiluminescence showed that WY-14643 diminished hypoxia-induced increase in intracellular ROS generation and depletion of UCP3 by siRNA abrogated the reduction of ROS with WY-14643 treatment (Figure 4(b)). For selective detection of mitochondrial superoxide, cells treated with 50 *μ*M of WY-14643 or vehicle were subjected to 20 h of hypoxia and MitoSOX Red-based flow cytometry was performed. Hypoxia increased mitochondrial superoxide generation (*P* < 0.05 versus control) and WY-14643 attenuated hypoxia-induced increase in mitochondrial superoxide (*P* < 0.05 versus hypoxia). In cells transfected with UCP3 siRNA, WY-14643 treatment did not reduce hypoxia-induced mitochondrial superoxide generation (*P* < 0.05 versus control, Figure 4(c)).

### 3.6. Cell Survival against Hypoxia-Reoxygenation Injury

**Figure 5:**
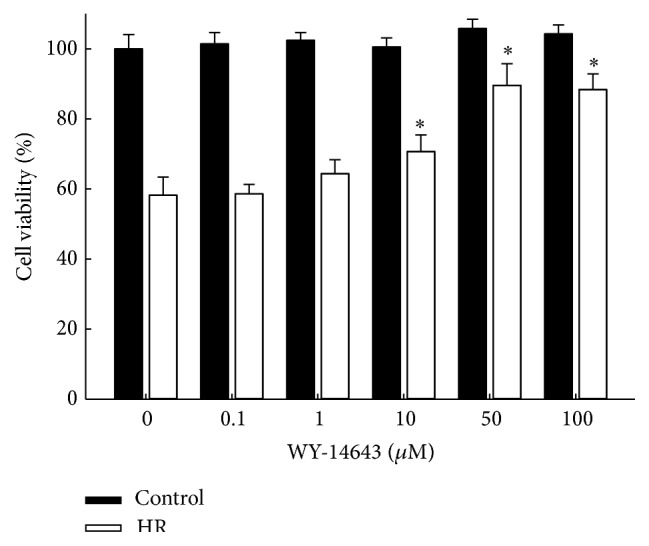
H9c2 cells treated with WY-14643 were more resistant against hypoxia-reoxygenation injury. Cells were incubated with medium containing 0 to 100 *μ*M of WY-14643 for 24 h before 20 h of hypoxia and 2 h of reoxygenation. MTT assay shows that cell viability was increased with treatment of WY-14643 10 *μ*M or greater. ^*∗*^ *P* < 0.05 versus WY-14643 0 *μ*M (*n* = 3). ^*∗*^ *P* < 0.05 versus WY-14643 0 *μ*M (*n* = 3). HR: hypoxia-reoxygenation.

**Figure 6:**
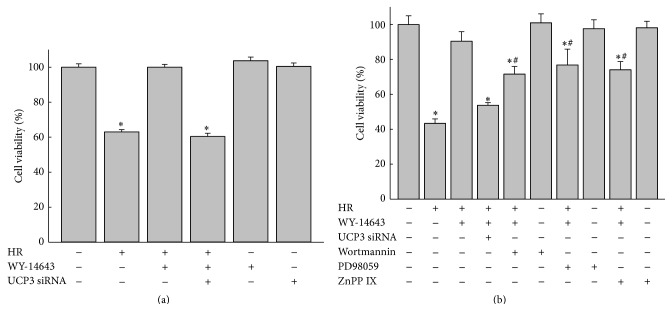
Depletion of UCP3 abrogated cell protective effect of WY-14643. Cells were incubated with medium containing 50 *μ*M of WY-14643 for 24 h before 20 h of hypoxia and 2 h of reoxygenation. (a) MTT assay shows that cell viability was increased with treatment of WY-14643 50 *μ*M while the enhanced viability was attenuated in cells transfected with UCP3 siRNA. ^*∗*^ *P* < 0.05 versus control (*n* = 3). (b) Wortmannin, PD98059, or zinc protoporphyrin-IX (ZnPP IX) decreased protective effect of WY-14643. Mitigated cell survival with treatment of Wortmannin, PD98059, or ZnPP IX was less significant compared with that of cells transfected with UCP3 siRNA. ^*∗*^ *P* < 0.05 versus control; ^#^ *P* < 0.05 versus HR (+), WY-14643 (+), and UCP3 siRNA (+) (*n* = 3). HR: hypoxia-reoxygenation.

Cell survival experiments were conducted to evaluate the protective effect of WY-14643 against hypoxia-reoxygenation (HR) injury. H9c2 cells incubated in control medium or experimental media treated with 0.1 to 100 *μ*M of WY-14643 for 24 h were subjected to 20 h of hypoxia and 2 h of reoxygenation prior to MTT assay. WY-14643 treatment at 10, 50, and 100 *μ*M decreased cell death after HR (*P* < 0.05 versus control, Figure 5). To examine the role of UCP3 in WY-14643 induced protection, cell survival experiment was also conducted in cells transfected with UCP3 siRNA. Cell protective effect of 50 *μ*M of WY-14643 against HR was diminished in cells transfected with UCP3 siRNA (*P* < 0.05 versus control, Figure 6(a)). Treatment of PI3 K inhibitor Wortmannin 100 nM, MEK inhibitor PD98059 50 *μ*M, or HO-1 inhibitor zinc protoporphyrin-IX 100 nM also decreased the protective effect of WY-14643 (*P* < 0.05 versus control). The degrees of decrease in cell survival following treatment with the above inhibitors were significantly less compared with that in cells transfected with UCP3 siRNA (*P* < 0.05 versus HR (+), WY-14643 (+), and UCP3 siRNA (+), Figure 6(b)).

## 4. Discussion

In the current study, administration of WY-14643, a PPAR*α* agonist, reduced myocardial infarct size following ischemia-reperfusion injury in rats. Treatment of WY-14643 in H9c2 cell line improved cell survival against hypoxia-reoxygenation. Activation of PPAR*α* by WY-14643 was associated with increased expression of UCP3* in vitro* and* in vivo*, which resulted in attenuation of ROS production. Furthermore, depletion of UCP3 by siRNA abolished the protective effect of WY-14643, implicating a critical role of UCP3 in PPAR*α*-mediated cardioprotection.

PPAR*α* is well known to be involved in myocardial fatty acid and glucose metabolism. It was demonstrated that cardiac specific PPAR*α*-overexpression increased myocardial fatty acid uptake and oxidation and concomitantly decreased glucose uptake and oxidation [2]. These metabolic alterations were associated with ventricular hypertrophy and contractile dysfunction, which was improved by low triglyceride diet [2, 4]. PPAR*α*-overexpressed mice also showed impaired recovery of myocardial function following ischemia-reperfusion injury [18]. These data suggest that chronic activation of PPAR*α* might be detrimental to cardiac function. On the contrary, acute activation of PPAR*α* by pharmacologic agonists demonstrated cardioprotection against ischemia-reperfusion injury [5–7, 19] while the underlying mechanisms or molecular targets involved in PPAR*α*-mediated cardioprotection have not been fully elucidated yet.

It was proposed that cardioprotection by PPAR*α* might be mediated through metabolic and anti-inflammatory effect. PPAR*α* agonist inhibited proinflammatory cytokine production and matrix metalloproteinase expression and activation of NF-*κ*B [5]. In accordance with the findings from PPAR*α*-overexpressed mice, increased fatty acid oxidation with concomitantly decreased glucose oxidation was also observed in rodents treated with PPAR*α* agonist compared with vehicle after acute ischemia-reperfusion injury [5]. Although elevated serum free fatty acid concentration is known to aggravate myocardial infarction [20], given that PPAR*α*-overexpressed mice showed systolic dysfunction with cardiac metabolic fuel shift, direct causal relationship between altered cardiac metabolism and cardioprotection against acute ischemia-reperfusion injury is still unclear. Other mechanisms including activation of the phosphatidylinositol 3-kinase/Akt and nitric oxide signaling pathway were also suggested [6, 7].

ROS is a critical mediator of ischemia-reperfusion injury [21]. Mitochondrial electron transport system is one of the major sources of cellular ROS generation. Return of oxygen supply during reperfusion induces large burst of ROS. Lipid peroxidation resulted from ROS breakdown of cell membranes. In addition, ROS trigger mitochondrial permeability transition pore opening, which causes loss of mitochondrial inner membrane potential and ceased ATP production and initiation of apoptosis [22]. Since mitochondria are major targets of damage from ROS as well as a source of production, reducing ROS burst during reperfusion may be important for maintaining mitochondrial function and cardioprotection. The production of ROS is proportional to mitochondrial membrane potential, and mild uncoupling of mitochondrial respiration is thought to reduce ROS production [23].

UCPs are inner mitochondrial carrier proteins that induce proton leak and dissipate the mitochondrial electrochemical gradient [8] and were suggested to exert a protective role during oxidative stress in previous studies. Indeed, UCP2 overexpression in rat neonatal cardiomyocyte increased viability against oxidative stress via reduced ROS production and mitochondrial Ca^2+^ overload [13]. Pharmacologic uncoupling agents such as dinitrophenol also conferred cardioprotective effect [11, 12, 24]. Recently, a crucial role of UCP3 in protection against myocardial ischemia-reperfusion injury was demonstrated in a cardiac UCP3 knockout mouse model [25]. Despite the fact that PPAR*α* regulate UCPs levels, the influence of PPAR*α* on UCPs and resultant ROS generation during myocardial ischemia-reperfusion has not been elucidated heretofore.

In the current study, treatment of WY-14643, a PPAR*α* ligand, upregulated the expression of UCP3, attenuated ROS production, and improved cell survival against hypoxia-reoxygenation. While WY-14643 also increased phosphorylation of kinases known to be involved in cardioprotective signaling pathways, protective effects of WY-14643 were nearly completely attenuated in cells transfected with UCP3 siRNA, which suggests that UCP3 may play a central role in cardioprotective effect of PPAR*α* activation. Since we have not shown whether knockdown of UCP3 also has an effect on phosphorylation of Akt and STAT3, it cannot be excluded that UCP3-induced cardioprotective effect of WY-14643 may involve the subsequent phosphorylation of cardioprotective kinases. As signaling ROS is known to be an important mediator in ischemic preconditioning [26] and upregulation of UCPs was also shown during ischemic preconditioning [14], cardioprotection by PPAR*α* activation may be associated with UCP-mediated mild uncoupling and subsequent increase in signaling ROS in resting cardiomyocytes.

Previous studies indicated that cardiac UCP3 level is regulated by PPAR*α* [15, 16]. In contrast to UCP3, UCP2 mRNA and protein levels were not affected by administration of PPAR*α* ligand in the current study. Normally, level of transcripts for UCP2 is more dominant than UCP3 in the rat heart [27]. The exact physiologic roles of UCP2 or UCP3 in intact heart are not yet completely understood. However, ectopic overexpression of UCP1 in mouse heart was also shown to improve the severity of ischemia-reperfusion injury [28]. Thus, it may be speculated that both UCP2 and UCP3, once activated during ischemia-reperfusion, can contribute to reduced ROS generation.

Reperfusion injury can be manifested by myocardial stunning or arrhythmia as well as myocardial infarction. ROS play a significant role in the development of myocardial stunning, a reversible contractile dysfunction following reperfusion [29] and arrhythmias [30]. Stability of the inner mitochondrial membrane potential is closely associated with reperfusion-induced arrhythmias [31]. Treatment of WY-14643 reduced the incidence of arrhythmia in the current study. In addition, there were trends towards higher mean arterial pressure and rate pressure product in rats that received WY-14643 during the early phase of reperfusion without statistical significance. Activation of UCP might have detrimental impact on myocardial contractility since increase in uncoupled respiration can lower mitochondrial ATP production. Indeed, increased UCP expression was observed in the failing human heart [32]. In acute ischemia-reperfusion, however, it might be possible that inefficiency in energy production is overcome with benefits of reduced oxidative stress.

Clinically, fibrates are synthetic PPAR*α* agonists widely used to treat dyslipidemia. Fibrates stimulate fatty acid oxidation, improve lipoprotein metabolism, and exert antiatherogenic effect by decreasing vascular inflammation via PPAR*α* activation [33]. Fibrates have been shown to be effective in prevention of cardiovascular events, particularly coronary disease [34]. Results of our study are in accordance with previous studies demonstrating beneficial effect of PPAR*α* activation and suggest a possible role of PPAR*α* agonists as a therapeutic agent for acute ischemia-reperfusion injury as well.

In conclusion, administration of PPAR*α* agonist WY-14643 decreased the degree of myocardial infarction and incidence of reperfusion-induced arrhythmia. PPAR*α* activation by WY-14643 conferred cytoprotective effect against hypoxia-reoxygenation. The current study also provides primary evidence regarding the underlying protective mechanism of WY-14643 that involves increased UCP3 expression and resultant attenuation of ROS production.

## Figures and Tables

**Table 1:** Hemodynamic data during ischemia-reperfusion.

	MAP (mmHg)		Heart rate (beats/min)		RPP (mmHg·beats/min)	
	Control	WY	Control	WY	Control	WY
Baseline	94 ± 15	107 ± 11	440 ± 40	450 ± 29	41438 ± 8847	48432 ± 6877
Before ischemia	97 ± 13	94 ± 24	435 ± 43	452 ± 42	42506 ± 7402	42428 ± 10754
Before reperfusion	89 ± 23^*∗*^	82 ± 24^*∗*^	436 ± 62	441 ± 76	39042 ± 12347	36719 ± 15041
15 min after reperfusion	77 ± 19^*∗*^	91 ± 27^*∗*^	401 ± 56	426 ± 57	31403 ± 11025^*∗*^	39381 ± 13750^*∗*^
30 min after reperfusion	77 ± 15^*∗*^	88 ± 15^*∗*^	419 ± 60	418 ± 62	32688 ± 9924^*∗*^	37592 ± 10024^*∗*^
60 min after reperfusion	75 ± 26^*∗*^	91 ± 15^*∗*^	419 ± 53	418 ± 60	32117 ± 13794^*∗*^	38494 ± 8261^*∗*^
2 h after reperfusion	87 ± 17	91 ± 22	425 ± 59	405 ± 43	37259 ± 9170^*∗*^	37268 ± 9514^*∗*^

Data are expressed as mean ± SD. ^*∗*^ *P* < 0.05 versus baseline.

MAP: mean arterial pressure; RPP: rate pressure product; WY: WY-14643 group.
